# Editorial to the *IJMS* Special Issue on Sglt2 Inhibitors Vol. 1

**DOI:** 10.3390/ijms24086873

**Published:** 2023-04-07

**Authors:** Anastasios Lymperopoulos

**Affiliations:** Laboratory for the Study of Neurohormonal Control of the Circulation, Department of Pharmaceutical Sciences, Nova Southeastern University Barry and Judy Silverman College of Pharmacy, Fort Lauderdale, FL 33328-2018, USA; al806@nova.edu; Tel.: +1-954-262-1338; Fax: +1-954-262-2278

The goal of this Special Issue is to highlight the ever-increasing progress in pharmacological research on sodium-glucose co-transporter (SGLT) type 2 (SGLT2) inhibitors or gliflozins. Originally developed for restoring euglycemia in diabetes mellitus, this drug class has increasingly been demonstrated to exert beneficial effects in patients, extending beyond blood glucose regulation. For instance, they reduce cardiovascular disease mortality, ameliorate kidney disease progression, lower blood pressure, and confer weight loss, even in non-diabetics or in people with end-stage chronic kidney disease. Indeed, during the time period between the opening of this Special Issue and its closing last year, several gliflozins were approved by the FDA for heart failure treatment with or without concomitant diabetes. In light of this background, this Special Issue of the International Journal of Molecular Sciences focused on studies that explore the biological and pharmacological effects of SGLT2i’s in human physiology and disease.

A few reviews published in this Special Issue focused on the already documented beneficial effects of SGLT2 inhibitors beyond glycemic control and diabetes mellitus. We provided a review with a research hypothesis on the sympatholytic effects of these drugs, as exemplified by dapagliflozin, as well as on the potential underlying molecular mechanisms for these effects [1]. We postulated that dapagliflozin downregulates adrenal G protein-coupled receptor kinase-2 (GRK2) and tyrosine hydroxylase (TH), both of which lead to reduced catecholamine synthesis and secretion from the chromaffin cells of the adrenal medulla [1]. At the same time, dapagliflozin stimulates the production of ketone bodies in the myocardium, such as beta-hydroxybutyrate, which can block the free fatty acid receptor type 3 (FFAR3), also known as G protein-coupled receptor-41 (GPR41), a receptor known to stimulate norepinephrine release from sympathetic neurons [2]. Thus, SGLT2 inhibitors may reduce sympathetic nervous system activity via both adrenal and cardiac sympathetic neuronal mechanisms. Another review explored the clinical evidence pertaining to SGLT2 inhibitors as well as the mechanisms underlying their kidney-protective effects [3]. SGLT2 inhibitors have been found to protect against kidney injury via glycemic control independently of blood glucose regulation, e.g., blood pressure lowering, hemodynamic correction, protection from lipotoxicity, and uricemic control [3]. Indeed, SGLT2 inhibitors are fast becoming part of the cornerstone pharmacotherapy of diabetic kidney disease.

In a detailed review, Trum et al. focused on the effects of SGLT2 inhibitors in heart failure with preserved ejection fraction (HFpEF) patients [4]. SGLT2 inhibitors may soon become the first class of approved drug to improve cardiovascular outcomes in this heart failure subpopulation. The proposed mechanisms for their benefits in HFpEF include metabolic and hemodynamic effects, as well as effects on inflammation, neurohumoral activation, and intracellular ion homeostasis [4,5]. Importantly, SGLT2 appears to be absent from the human adult myocardium; therefore, exactly how SGLT2 inhibitors directly benefit the myocardium remains a molecular mystery. The review focused on the growing body of evidence for SGLT2 inhibitors that affect cardiac intracellular Na^+^ as a molecular mechanism, i.e., an SGLT2-independent mechanism [4,5]. The authors provided an overview of physiological cardiomyocyte Na^+^ handling and its deterioration in heart failure, and then proceeded to describe the salutary effects of the gliflozins on Na^+^ homeostasis by influencing sodium/proton exchanger (NHE)-1 activity, late sodium currents, and calcium/calmodulin-dependent kinase II (CaMKII) activity [4]. Given the aforementioned controversy surrounding the absence of myocardial SGLT2, Sayour et al. reviewed the effects of dual SGLT1/2 inhibitors, such as sotagliflozin, and explored the additional benefits afforded by SGLT1 inhibition [6]. In addition, the authors elegantly demonstrated the clinical significance of myocardial SGLT1 inhibition and how this blockade might factor into the clinical benefits of SGLT2 inhibitors [6].

Finally, a couple of reviews included in the Special Issue discussed the vascular effects of SGLT2 inhibitors [7] and their safety from a pharmacogenetic standpoint [8]. Regarding the vascular benefits and mechanisms of SGLT2 inhibitors, these drugs enhance the bioavailability of endothelium-derived nitric oxide, restoring endothelium-dependent vasodilation in diabetes [7]. In addition, SGLT2 inhibitors favorably regulate the proliferation, migration, differentiation, survival, and senescence of vascular endothelial cells [7]. Moreover, they exert potent antioxidant and anti-inflammatory effects in the vessel wall endothelium [7]. SGLT2 inhibitors can even inhibit vascular smooth muscle contraction and cell proliferation, thereby exerting protective effects against post-angioplasty restenosis, maladaptive vascular remodeling (underlying pulmonary arterial hypertension—PAH), abdominal aortic aneurysm development, and diabetes-associated arterial stiffness [7].

The Special Issue also features a few interesting and original research articles on novel effects of SGLT2 inhibitors in renal tubular and vascular endothelial cells. Shirakawa and Sano investigated the molecular mechanisms of SGLT2 inhibitors’ renoprotective effects by examining the transcriptional activity of osteopontin, a key mediator of renal and cardiac fibrosis [9,10]. They found that high-glucose conditions increased SGLT2- and GLUT-mediated glucose uptake, leading to the formation of elevated mitochondrial reactive oxygen species (ROS) and transcriptional activation of osteopontin [9]. Importantly, canagliflozin blocked the overexpression of myoinositol oxygenase and prevented aberrant glycolytic metabolism, as well as mitochondrial ROS formation in proximal tubular cells on high glucose [9]. Empagliflozin, on the other hand, was shown by Pirkbauer et al. to attenuate the expression of several pro-inflammatory response genes in normoglycemic human proximal tubular cells treated with interleukin-1β (IL-1β) [11]. Upon a comprehensive transcriptomic analysis, these authors were able to identify novel genes, such as CXCL8/IL8, LOX, NOV, PTX3, and SGK1, that may be causally involved in glycemia-independent nephroprotection by SGLT2 inhibitors such as empagliflozin [11]. Additional mechanistic evidence for the renoprotective effect of empagliflozin was provided by Huang et al., who showed that empagliflozin attenuated high-fat diet (HFD)-induced body weight gain, insulin resistance, and inflammation in mice via the downregulation of renal tubular CD36, a class B scavenger receptor that mediates free fatty acid (FFA) uptake in renal proximal tubular cells. CD36 was upregulated in the tubular area of the kidney, whereas empagliflozin attenuated CD36 expression [12]. The authors went on to show that empagliflozin induces CD36 downregulation via peroxisome proliferator-activated receptor (PPAR)-γ modulation [12]. Thus, empagliflozin can ameliorate FFA-induced renal tubular injury via the PPARγ/CD36 pathway [12] (Figure 1). Nevertheless, an interesting and provocative study by Zhang et al. suggested that blood glucose levels per se do not alter glucose influx or efflux kinetics in renal proximal tubules since the SGLT2 inhibitor luseogliflozin was not found to exert significant effects on glucose influx parameters under any level of blood glucose concentration [13]. The authors speculated that this was because glucose influx occurs through basolateral GLUT2, which is not a direct target of SGLT2 inhibitors [13].

Apart from the kidney, the effects of SGLT2 inhibitors in cardiovascular cell types were also examined in a few other studies. Li et al. examined the effects of three SGLT2 inhibitors—empagliflozin, dapagliflozin, and canagliflozin—on anti-oxidative protection and cyclic stretch-induced endothelial permeability in human coronary artery endothelial cells (HCAECs) [14]. All three drugs improved the barrier dysfunction of HCAECs under enhanced stretch via the scavenging of ROS [14] (Figure 1). In addition, the anti-oxidant actions of the three SGLT2 inhibitors were partly mediated by the inhibition of NHE1 and various isoforms of nicotinamide adenine dinucleotide phosphate oxidase (NOX) [14]. Canagliflozin was also found to ameliorate maximal vasorelaxation to acetylcholine in ischemia/reperfusion (IR)-subjected rat aortic rings [15] (Figure 1). Mechanistically, canagliflozin significantly reduced IL-1α and IL-6 mRNA expressions. Both genes were upregulated by IR, downregulating NoxO1 gene expressions, decreasing intercellular adhesion molecule (ICAM)-1 and nitrotyrosine levels, and increasing platelet and endothelial cell adhesion molecule (PECAM)-1 immuno-reactivity [15]. Thus, canagliflozin (and potentially other SGLT2 inhibitors) alleviates endothelial dysfunction following IR injury. Finally, the anti-arrhythmic potential of empagliflozin in murine ventricular tissue was examined by Jhuo et al. [16]. Compared with HFD-fed mice, empagliflozin significantly shortened the QT interval and effective refractory period (ERP) of the left ventricle [16]. Empagliflozin also attenuated connexin (Cx)-40 and Cx43 downregulation induced by HFD, an effect not shared by the oral hypoglycemic agent glibenclamide. Importantly, reduced the number of fibrotic areas of the HFD-fed murine ventricles [16] (Figure 1). Therefore, empagliflozin may protect against ventricular arrhythmias induced by a high-fat diet. Given that empagliflozin has the highest specificity for SGLT2 among the FDA-approved SGLT2 inhibitor drugs, it will be intriguing to see in future studies whether the anti-arrhythmic effect of empagliflozin is shared by other SGLT2 inhibitors.

## Figures and Tables

**Figure 1:**
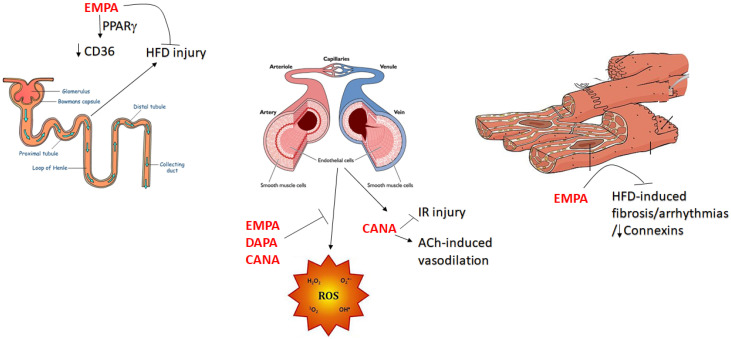
Protective effects of SGLT2 inhibitors in renal cells (**left**), vascular (coronary and aortic) endothelial cells (**middle**), and ventricular myocytes (**right**), as reported in this Special Issue [12,14,15,16]. See text for details. EMPA: Empagliflozin; DAPA: Dapagliflozin; CANA: Canagliflozin; ACh: Acetylcholine.
